# Comparing Forests across Climates and Biomes: Qualitative Assessments, Reference Forests and Regional Intercomparisons

**DOI:** 10.1371/journal.pone.0094800

**Published:** 2014-04-17

**Authors:** Carl F. Salk, Ulrich Frey, Hannes Rusch

**Affiliations:** 1 Institute of Philosophy, University of Giessen, Giessen, Germany; 2 University of Colorado Institute of Behavioral Science, Boulder, Colorado, United States of America; 3 International Institute for Applied Systems Analysis, Laxenburg, Austria; 4 Behavioral Economics, University of Giessen, Giessen, Germany; 5 Peter Löscher Chair of Business Ethics, Technical University of Munich, Munich, Germany; The Ohio State University, United States of America

## Abstract

Communities, policy actors and conservationists benefit from understanding what institutions and land management regimes promote ecosystem services like carbon sequestration and biodiversity conservation. However, the definition of success depends on local conditions. Forests' potential carbon stock, biodiversity and rate of recovery following disturbance are known to vary with a broad suite of factors including temperature, precipitation, seasonality, species' traits and land use history. Methods like tracking over-time changes within forests, or comparison with “pristine” reference forests have been proposed as means to compare the structure and biodiversity of forests in the face of underlying differences. However, data from previous visits or reference forests may be unavailable or costly to obtain. Here, we introduce a new metric of locally weighted forest intercomparison to mitigate the above shortcomings. This method is applied to an international database of nearly 300 community forests and compared with previously published techniques. It is particularly suited to large databases where forests may be compared among one another. Further, it avoids problematic comparisons with old-growth forests which may not resemble the goal of forest management. In most cases, the different methods produce broadly congruent results, suggesting that researchers have the flexibility to compare forest conditions using whatever type of data is available. Forest structure and biodiversity are shown to be independently measurable axes of forest condition, although users' and foresters' estimations of seemingly unrelated attributes are highly correlated, perhaps reflecting an underlying sentiment about forest condition. These findings contribute new tools for large-scale analysis of ecosystem condition and natural resource policy assessment. Although applied here to forestry, these techniques have broader applications to classification and evaluation problems using crowdsourced or repurposed data for which baselines or external validations are not available.

## Introduction

Secondary, degraded, managed and other human-impacted forests are of increasing abundance worldwide [1], [2]. Regenerating forests are recognized as increasingly important reservoirs of biodiversity and are of central importance to carbon sequestration projects such as REDD+ (Reduced Emissions from Degradation and Deforestation). As such, it is an urgent priority to understand which management regimes, including the social and political context of forest management, lead to desired forest outcomes. Unfortunately, measuring forest structure or biodiversity is difficult and is further complicated when forests with different underlying conditions (e.g. climate, species pools, land-use histories) are compared. Two successional forests starting regeneration at the same time cannot be expected to show the same trajectories of structure and biodiversity if underlying environmental conditions are different, even if they are both managed as untouched reserves.

Forests differ from one another due to many locally variable environmental and social circumstances, making direct comparisons of most forest-related variables among sites misleading. Local factors can affect the total possible biomass and biodiversity at a site, the rate at which these totals are reached and the starting point for forest structure and biodiversity trajectories. Thus, two forests with similar management institutions may have different patterns of forest structure or biodiversity simply from underlying environmental or historical differences. Similarly, two forests with equal species richness or basal area may represent completely different ecological outcomes in the local context of potential species pools, tree growth rates and management history.

Site-level potential biomass has numerous known environmental correlates. Forests grow faster and attain a higher stature in warmer climates and where seasonal temperature differences are small, although these effects are difficult to disentangle from soil- and cloudiness-related processes [3]. Potential forest biomass tends to be higher on nutrient rich soils [4]. While difficult to test experimentally, traits of species present in a site, particularly wood density, also affect total potential biomass [5]. Forest successional trajectories are also environmentally dependent. Forest productivity increases strongly with annual precipitation in dry to moderate climates, but decreases somewhat in very wet sites [6]. Forest biomass accumulates faster on soils with more nutrients [7].

Total potential biodiversity increases with total rainfall and seasonality within the tropics [8]. The dramatic difference between tropical and temperate forest biodiversity is well known, although not fully explained [9]. Plant diversity usually increases with temperature [10]. Wetter and less seasonal tropical forests have more species than are found in sites with less rainfall [11] or a more pronounced dry season [12]. Tree biodiversity may increase with soil fertility [11], [12] but see [13]. The number of tree species found per hectare of forest declines with latitude [11] and altitude [14], although these patterns are almost certainly artifacts of covariation with factors like temperature [15], or historical and evolutionary processes [16], [17]. While the study of biodiversity accumulation through succession is an emerging field with many unanswered questions, it is a process that depends on many environmental covariates [18]. Finally, both biomass and diversity of a forest depend on the state it was in when successional processes began.

Comparing biodiversity across sites is further complicated by the inevitable but non-linear increase in species richness and most other biodiversity metrics with area sampled. On top of this problem, the number of tree species found per area is influenced by stem density; in aggregate, stands with more stems per hectare also have more species per hectare [19], [20].

Previous researchers have addressed these difficulties by evaluating forests against nearby old-growth forests or by assessing the trajectory of individual forests' changes. Tucker and colleagues [21] used “reference forests”, that they define as “old-growth forests … relatively undisturbed by natural and human influences.” Managed forests were compared against the reference state using four equally-weighted indices. Three of these indices measure structural characteristics of the forest: (1) basal area (BA) per hectare for trees ≥10 cm diameter at breast height (DBH), (2) the mean DBH of trees ≥10 DBH and (3) the ratio of number of stems per hectare ≥10 cm DBH to the total number of stems ≥2.5 cm DBH. The fourth index was a count of the total number of tree species (≥10 cm DBH) encountered in a site's plots, not corrected for area or number of stems sampled. Because raw species counts necessarily increase with number of stems sampled, they are not comparable, even among environmentally similar forests. Nagendra [22] used a change over time approach to assess the relative trajectory of forest structural attributes (e.g. stem density, average DBH, tree height) and species richness. That study compared plot-derived estimates of changes in these metrics for forests visited at least twice with assessments of these same metrics by forest users (a group that was defined very broadly and included separate discussions with men and women and other possible divergent views) and expert foresters. While many individual cases showed disagreements, there was a general agreement among assessments from plot-based data, users and foresters, although users had a better agreement with plot-based data than did foresters.

Environmental and historical differences among sites make it clear that forest comparisons of forest management success must account for local differences in potential forest structure and biodiversity. The success of forest management is typically measured against old-growth and minimally impacted forests [21]. However, even this reference state can be misleading because attributes like basal area and biodiversity can overshoot their old growth levels in mid-successional forests [23], [24] and in any case may not be a relevant measure of forest management success.

In this study we compare several methods of forest assessment, including changes over time, comparison with reference forests and a new method we introduce of “regional intercomparison” of multiple nearby stands. Further, we refine the reference forest concept, focusing on basal area and biodiversity for their direct relevance to key ecosystem services of carbon sequestration and biodiversity conservation and we adopt methods from the ecological literature to improve species richness estimates. We apply these methods to several hundred managed forests from a world-wide database. The results show that most methods give broadly congruent results, suggesting that social scientists and conservation practitioners have substantial flexibility in assessing forest conditions, even in the absence of reference forests or baseline data.

## Methods

### Data

The forests compared in this study come from the International Forestry Resources and Institutions (IFRI) research network. The IFRI collaboration is a trans-national comparison of success factors in common pool resource governance initiated by Elinor Ostrom in the early 1990s. IFRI has a decentralized structure, with individual sites' data contributed by in-country collaborating research centers. The database contains hundreds of variables on institutions, human demography and forests [25]. This study uses forest data measured directly in plots and ordinal assessments of forests by local users and foresters with regional expertise. Most IFRI sites are in Latin America, east Africa, south Asia, or the Midwestern USA. This analysis uses virtually all IFRI sites available as of 2011, excluding only a few forests which have no extractive wood use. Previous studies have used smaller subsets of IFRI sites to compare forests using some of the methods addressed here, including reference forests [21] and user and expert evaluations [22]. Certain analyses were only possible for subsets of forests for which appropriate data (such as reference forests, or site revisits) was available. The total analysis covers 297 forests, 84 of which have baseline data from previous visits. See Table 1 for further details on regional distribution of sites.

**Table 1:** **Summary of forests analyzed in this study.**

Location		Forests (revisits)	Years	Forest types
*Americas*	Bolivia	19 (3)	1994–2008	Tropical lowland, Subtropical montane
	Brazil	2	1996–1997	Tropical lowland
	Colombia	1	2001	Tropical montane
	Ecuador	1	1995	Tropical lowland
	Guatemala	18 (4)	1996–2007	Tropical lowland, Tropical montane
	Honduras	7	1997–1998	Tropical montane
	Mexico	12 (5)	1999–2008	Tropical montane
	United States	6 (5)	1995–2008	Temperate lowland
*Africa*	Kenya	15 (11)	1997–2007	Tropical lowland, Tropical montane, Savanna, Mangrove
	Madagascar	10	1997–1999	Tropical lowland, Spiny thicket
	Tanzania	8	1998–2003	Tropical lowland, Tropical montane, Mangrove
	Uganda	35 (29)	1993–2008	Tropical lowland, Tropical montane
*Asia*	Bhutan	6	2001	Subtropical montane
	India	45 (8)	1993–2007	Tropical lowland, Subtropical montane
	Nepal	108 (23)	1993–2008	Subtropical montane
	Thailand	3	2003–2007	Tropical lowland

The number of forests outside of parentheses is the total number from each location analyzed in this study. The number in parentheses is the number of those forests that had been previously visited. The “years” column indicates the earliest and latest years in which site visits in the database occurred.

IFRI forest measurements are made on circular plots randomly located within each forest. Within 10 m of each plot center, the diameter at breast height (DBH) of all trees (including palms) ≥10 cm DBH is measured and recorded and all such trees are identified to species. Within 3 m of the plot center all tree saplings between 2.5 and 10.0 cm DBH are identified and measured, as are all shrubs and lianas ≥2.5 cm DBH. Data from trees and saplings was used for forest structure calculations (shrubs and lianas were omitted because of problems with measurement consistency) and all life forms were used in biodiversity assessments. One tree with an obviously anomalous DBH measurement (18.37 m) was omitted from the basal area analysis. Because two spellings of the same species appear to be two different species to a computer, inflating species counts, we individually reviewed species' spellings to ensure that within a site, each species was spelled consistently.

The IFRI database contains several variables on users' and foresters' assessments of forests. We use four of these for comparisons with plot-based methods. Two of the questions are addressed to users of the forest. The first of these (variable *ΔD_user_* in this paper) is “Has the density of trees on the forest land changed in the past five years?” The second (*ΔA_user_*) is “During the last five years, has there been any change in the area over which vegetation exists/existed?” These questions have three possible answers - decrease, no change and increase - that were coded as −1, 0 and 1 for analysis. Two additional questions ask a professional forester to assess “The density of vegetation in this forest” (*D_forester_*) and “The species diversity in this forest” (*SR_forester_*) in the context of “the topography and ecological zone in which this forest is located.” The vegetation density question, as worded in the IFRI manual, is quite vague and should not be assumed to be equivalent to stem density, although some respondents may interpret it that way. Other interpretations of this question might include density of undergrowth, percent canopy cover, or amount of timber or biomass available. These questions have five possible answers: very low, low, normal, high and very high that were coded as −2, −1, 0, 1 and 2 respectively. For a summary of these variables, see Table 2 and the IFRI manual [25].

**Table 2:** **A list of all variables used in this paper.**

Variable	Category	Description
*ΔBA*	Forest structure	Annualized change in basal area since the last forest visit
*BA_int_*	Forest structure	Basal area per hectare regionally intercompared with other IFRI forests
*BA_ref_*	Forest structure	Basal area per hectare relative to nearby reference forests
*ΔD_user_*	Forest structure	Users' appraisal of change in tree density over the last 5 years (IFRI variable FTREEDENS)
*D_forester_*	Forest structure	Foresters' appraisal of forest vegetation density (IFRI variable FVEGDENSE)
*ΔA_user_*	Forested area	Users' appraisal of change in forested area over last 5 years (IFRI variable FVEGCHANGE)
*ΔSR*	Biodiversity	Percent change in rarefied species richness per year
*SR_int_*	Biodiversity	Rarified species richness regionally intercompared to nearby IFRI forests
*SR_ref_*	Biodiversity	Rarefied species richness relative to nearby reference forests
*SR_forester_*	Biodiversity	Foresters' appraisal of forest species diversity (IFRI variable FSPECIEDIV)

For details of how variables were calculated, see the main text. When a variable comes directly from the IFRI database, the name of that IFRI variable is given in all caps.

### Basal area calculations

Basal area (hereafter BA) was chosen as the metric by which to compare forest structure. Other forest structure calculations, particularly biomass per hectare, hold the appeal of being directly relatable to ecosystem-level processes, particularly stocks and flows of carbon and related applications (e.g. REDD+). However, the allometric equations for tree biomass assume round trunks as a starting point and build additional uncertainty from there. Further, wood density varies over about two orders of magnitude [26]. While species-specific allometries and wood density estimates are available for some species, their application would be onerous and only a partial improvement. If these species-specific corrections are not used, tree biomass equations are typically second order polynomials of tree diameter [27], making biomass well correlated with basal area [28].

BA per hectare for IFRI sites was computed from plot-based IFRI tree inventory data, by entering a tree's measured DBH into the formula for the area of a circle (A = π·DBH^2^/4), summing these values over a forest's plots and dividing by the plots' total area. It was calculated separately for trees and saplings in each visit's forest plots. This was necessary because the sapling (stems ≥2.5 cm) and tree (stems ≥10 cm) plots had different areas (28.3 m^2^ and 314 m^2^, respectively). The per-hectare tree (including palms) and sapling basal areas were summed to a provide total value for each forest-visit. Non-tree species (vines and shrubs) were not included. Data from one set of sites in India used different sized plots (5 m radius for both trees and saplings) and measured tree circumferences rather than diameters, so we corrected their basal area and biodiversity calculations for these differences. Since the reference forest data (see below) includes measurements of all stems ≥2.5 cm in a 1000 m^2^ plot, it was not necessary to separate out stems into size classes for the basal area computations. For comparability with the IFRI data, non-tree life forms were not included in these calculations.

### Biodiversity calculations

Biodiversity is hard to estimate in diverse tropical forests [28]. It can be calculated on a per-area or per-stem basis. Because area-based biodiversity metrics are highly sensitive to stem density [29] and stem density varies widely with attributes such as climate, forest age, and disturbance level, we use stem-based metrics. The total number of species is an increasing but non-linear and concave-down function of sampling effort (whether measured in terms of area or stems), so steps must be taken to compare among heterogeneous datasets.

Biodiversity was made more comparable among sites by estimating median species richness (SR) for a sample of 100 stems including all growth forms (trees, saplings, vines/lianas and shrubs). For each forest-visit, all stems were pooled. From this pool 100 stems were randomly sampled with replacement and the number of unique species encountered was counted. This was repeated 1000 times to generate a distribution from which the median rarefied species richness was computed, referred to here as “SR”. The choice of 100 as the number of stems sampled was a tradeoff between having enough samples to see real variability among forests and sampling a number that was less than the number of stems in most forests, avoiding undercounts due to sampling rather than ecology. This final assumption was met in 87% of forests with plot data. Those with fewer than 100 stems were mostly heavily harvested, with few standing trees and thus of genuinely low biodiversity.

### Change over time comparisons

The most basic calculations of forest condition were how BA and SR changed over time in forests that had been re-visited. Absolute changes depend both on the starting condition of the forest and the amount of time between measurements. We compensated for these factors by calculating the annualized relative change in BA as 
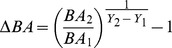
where *BA_1_* and *BA_2_* are the observed basal areas in years *Y_1_* and *Y_2_* respectively. This variable has a value of 0 if BA remains unchanged; positive values indicate growth and negative values mean decreasing basal area. An analogous value of *ΔSR* was also calculated. Note that these metrics reflect absolute change in BA or SR and do not address more subtle changes such as altered size distributions of trees or turnover of species present.

### Regional intercomparison

As an alternative to reference forests, we devised a new method to compare BA and SR of each forest with nearby forests from the same dataset, a technique we call “regional intercomparison.” For each IFRI forest, we computed the geographic distance (in km) and elevation difference (in m) to each other IFRI forest. Geographic distances were calculated as great circles by applying the haversine formula to the sites' coordinates and multiplying by the radius of the earth (6371 km). Geographic and elevation distances were combined into a “total distance” (TD) between site-pairs, taking into account both geographical and altitude separation, by summing the squares of both types of distance and taking the square root. Note that the units on these two distances were not the same, so the “total” distance does not have meaningful units, but gives an effective equivalency between 1 m altitude and 1 km distance. This equivalency is based on the approximate similarity between changes in temperature seen in a climb of 1 km or an 800 km poleward journey at middle latitudes [30]. A forest was considered nearby if it was within 1000 of these total distance “units” of the focal forest. The cutoff of 1000 units was chosen to reflect the spatial clustering typically found in IFRI countries. In most cases, only sites much closer than 1000 units influenced these calculations because of distance-based weighting (see following paragraph).

A z-score (i.e. a value rescaled to a normal distribution with mean 0 and standard deviation of 1) was calculated for BA and SR for each forest. The underlying parameters for these normal distributions were computed as the weighted mean and standard deviation for BA and SR for all nearby IFRI forests. As weights, TD^−2^ was used to weight closer forests more heavily. BA and SR values normalized relative to IFRI forests using this regional intercomparison method are referred to as *BA_int_* and *SR_int_*.

### Comparisons with reference forests

As reference forest data, we used a database of old-growth forest plots collected by Alwyn Gentry [11]. This database has worldwide coverage, with best representation in the Western Hemisphere tropics. These plots cover .1 ha each, using 10 parallel transects, each 50 m long and 2 m wide. Within each transect, each stem >2.5 cm DBH was measured and identified to species.

Comparisons of IFRI forest BA with reference forests were done as described above for IFRI forest intercomparison, except pairwise distances were calculated between the focal IFRI forest and nearby reference forests, rather than among IFRI forests. The distance cutoff of 1000 units was applied in the same way as for reference forests.

Because of differences between IFRI and reference forest sampling techniques, SR was recalculated to make these data directly comparable. While the stem-based sampling method renders sites with different extents of forest surveys more comparable, it is not able to erase large differences in sampling effort, such as those between the reference forests (.1 ha sampled) and a typical IFRI forest where ∼1 ha was sampled. To solve this problem, each IFRI forest-visit's plots were resampled 1000 times, each time choosing 3 plots, without replacement. This gave a total area sampled of .0942 ha, very similar to the .1 ha area of the reference forest samples. In the case of forests in India with 5 m radius plots, we used 12 plots for tree and palm biodiversity, that cover exactly the same area as in 3 standard sized IFRI plots. For other lifeforms, we used one randomly chosen plot, for a total of 78.5 m^2^, an area quite similar to that of 3 standard (3 m radius) understory plots (84.78 m^2^). For each of these subsets of plots 100 trees were sampled with replacement to estimate a median value of SR.

A similar correction was made for different sampling of small stems. In the reference forests all stems greater than 2.5 cm were sampled throughout the plots, while in the IFRI forests stems between 2.5 and 10 cm are only sampled in a 3 m radius subplot (except in the sites from India in which they were sampled over the entire 5 m radius plot). Thus, the reference forests represent a much greater effort to sample smaller stems, likely inflating the count of species that do not typically reach a large size or have not yet grown large due to successional changes in the forest. This problem was solved by using only part of the reference forest data on stems <10 cm DBH in each repetition of the SR_100_ sampling on the reference forest data. Each reference forest plot is composed of 10 subplots of 100 m^2^ each. In each SR sampling repetition one of these reference forest subplots was randomly chosen for inclusion of saplings into the total population of stems; plants <10 cm DBH from the other nine subplots were ignored. This 100 m^2^ sampling area is similar to the 85 m^2^ ( = 3 * π(3 m)^2^) sampled for saplings in 3 IFRI plots.

**Figure 1:**
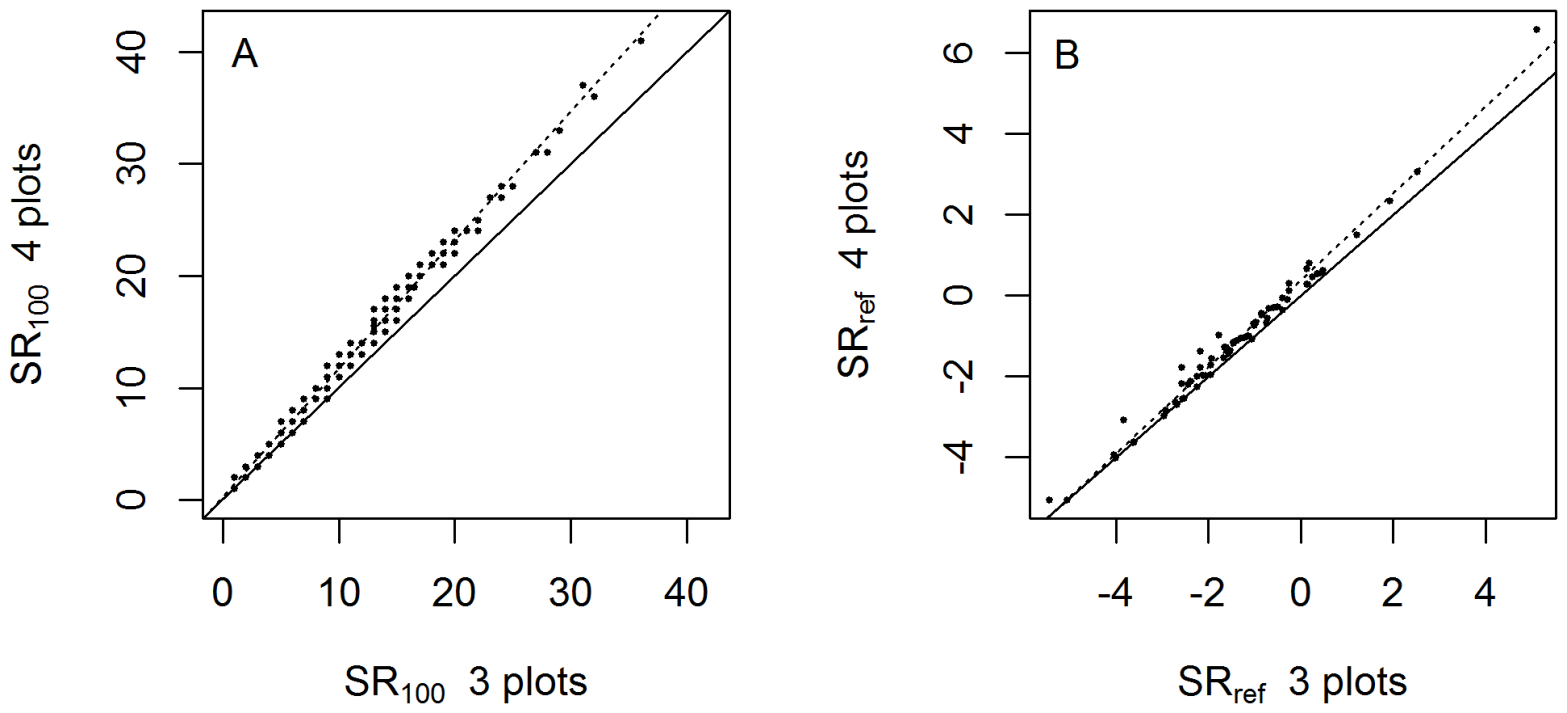
Biodiversity metrics as computed using 3 vs. 4 IFRI plots. A) Median species richness in a 100 stem sample (*SR_100_*) based on samples of 3 and 4 plots. B) Median species richness in a 100 stem sample relative to nearby old-growth reference forests (*SR_ref_*), as calculated with samples of 3 and 4 plots. In both panels, the solid line is a 1∶1 line and the dotted line is a major axis (model II) regression line.

The safety of both of these assumptions was confirmed by re-running the sampling as described above, but with 4 IFRI plots for a total area of .1256 ha (trees) and .0113 ha (saplings) to bracket the area covered by one Gentry plot (trees) and one Gentry subplot (saplings). Estimated biodiversity per 100 stems sampled was slightly greater when the sampling drew from a 4-plot pool rather than only 3 plots (Figure 1A). When the 4 plot biodiversity estimates were used to compute biodiversity relative to Gentry plots, the z-scores were barely higher. After rescaling, Gentry-relative biodiversity values were quite comparable regardless of whether 3 or 4 IFRI plots were used in the calculations (Figure 1B). The correlation between the two scaled values had an R^2^ = .99 and regression coefficients very close to intercept 0, slope 1.

### Statistical methods

Forest variables were compared within two groups, one for forest structure (*ΔBA*, *BA_int_*, *BA_ref_*, *ΔD_user_*, *D_forester_*) and a second for biodiversity (*ΔSR*, *SR_int_*, *SR_ref_*, *SR_forester_*). Within each group the relationship between each pair of variables was assessed as described below. Because the users' appraisal of forested area changes (*ΔA_user_*) could be related to either forest structure or biodiversity changes, this variable was compared with all other variables. To test the independence of estimates of different forest attributes, we also compared each forest structure variable with each biodiversity variable and vice versa.

Because of the unique advantages and drawbacks of each method of assessing forests, it does not make sense to assume that any of these methods is better or less error prone than others as one would in order to assign independent and dependent variables. Thus, ordinary least squares regression, which assumes error only in the dependent variable, is not appropriate for this analysis. Instead, we used major axis (model II) regression [31], as implemented in the R package *lmodel2* (http://cran.r-project.org/web/packages/lmodel2/index.html). Unlike ordinary least squared regression, when the identity of the independent and dependent variables is switched type II regression returns the same relationship between the variables.

Ordinal variables (i.e. users' and foresters' estimates of forest attributes) were assessed against continuous variables using ANOVAs, implemented with the R function aov(). ANOVAs were also used when two ordinal variables were compared with one another. In these cases, the analyses were done both ways, i.e. with the identities of the independent and dependent variables switched, with very similar results. Treating both variables as continuous (regression) also gave similar, but less nuanced, results (not shown).

## Results

### Forest structure

**Figure 2:**
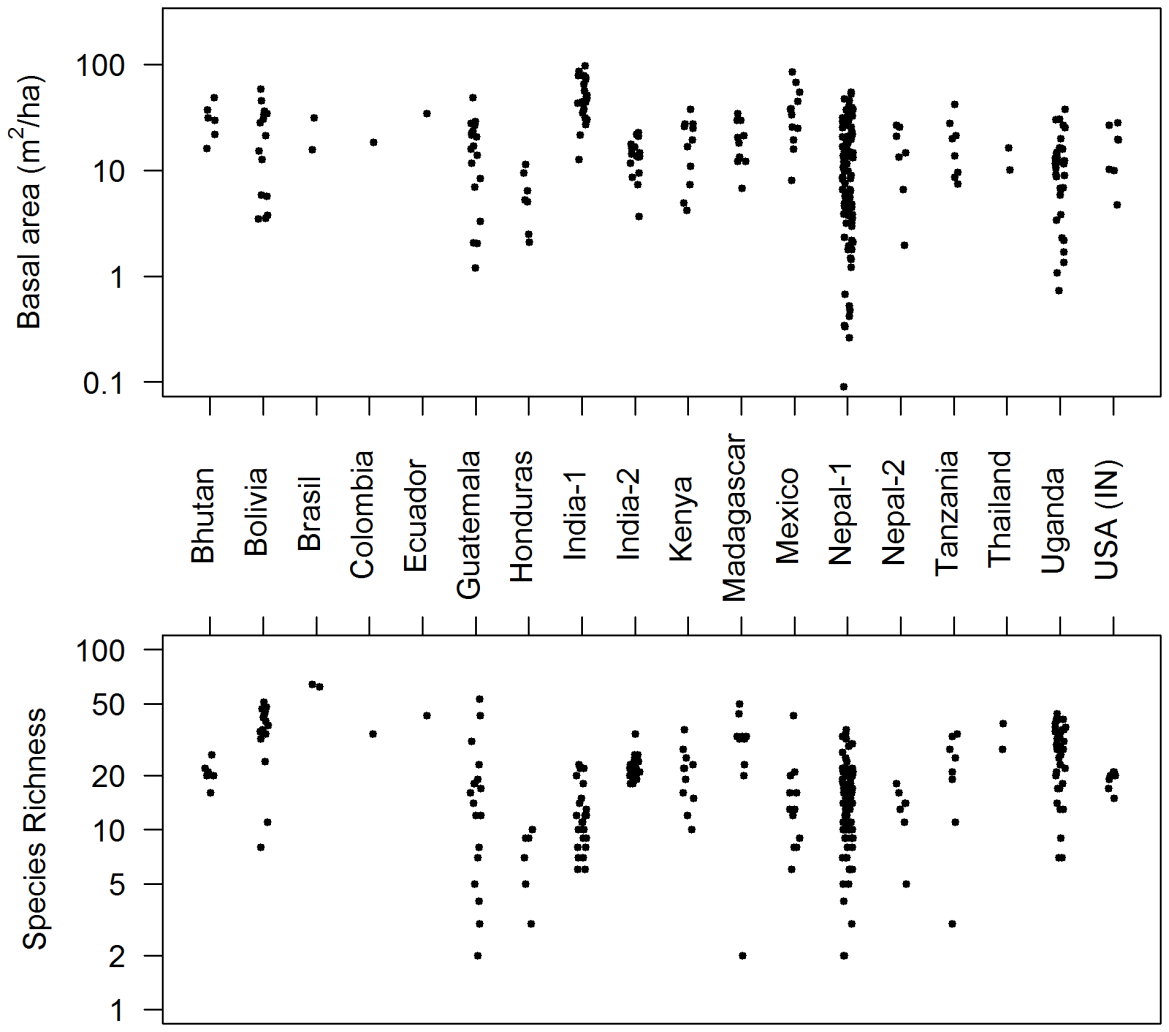
Basal area and species richness values for all sites included in this study by country. In cases where there are two different columns for a country, there were two different groups working in that country, typically in different regions. All USA sites are in the state of Indiana. Species richness values presented here are not raw, but are corrected for number of stems sampled. (See main text for details.)

**Figure 3:**
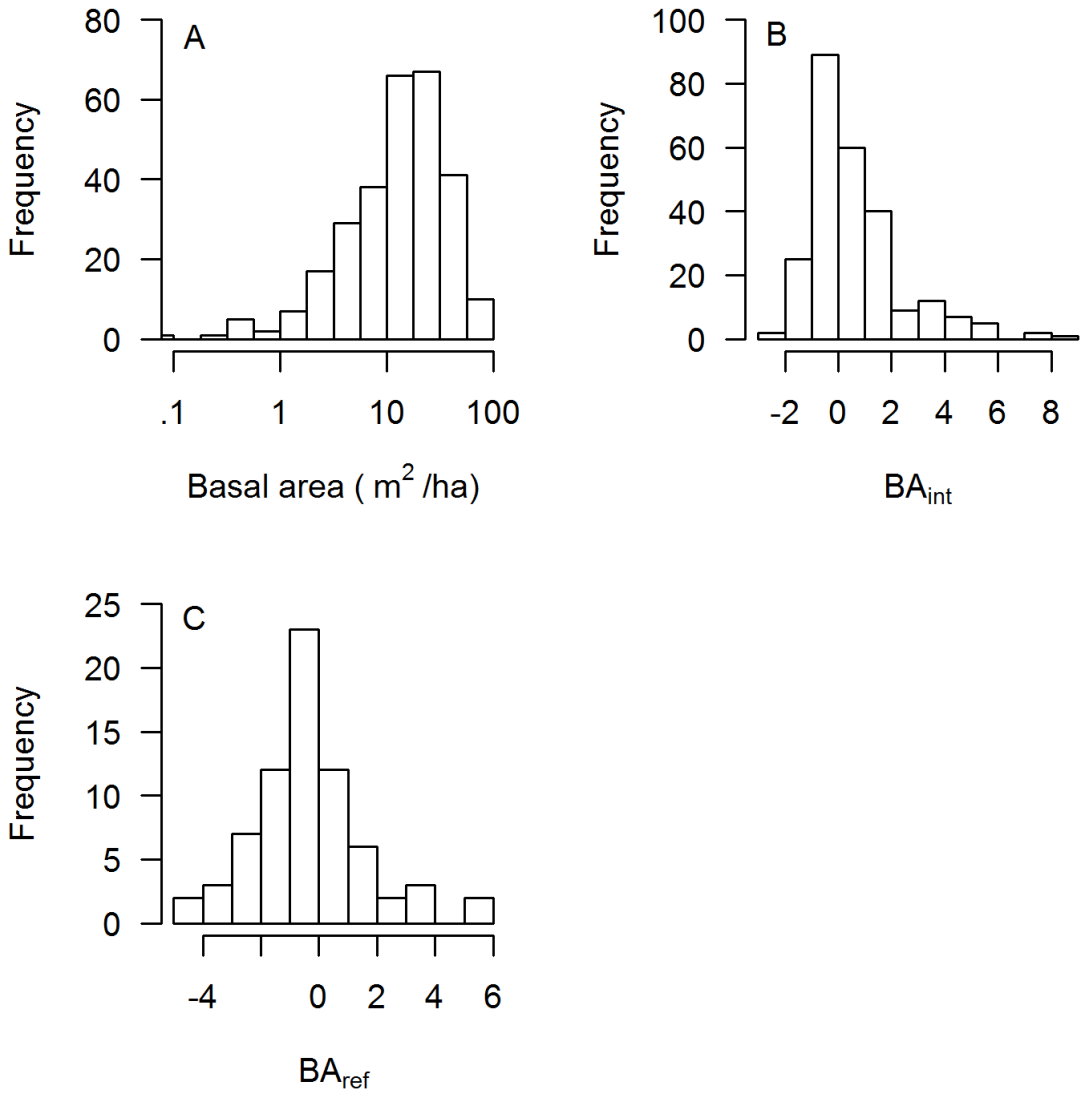
Frequency distributions of forest-level plot-derived basal area at sites used in this study. A) Raw basal area (note x-axis is on a log scale). B) Basal area normalized by regional intercomparison to nearby forests in the same database. C) Basal area normalized relative to nearby reference (mature) forests. (See text for details of these calculations.)

**Figure 4:**
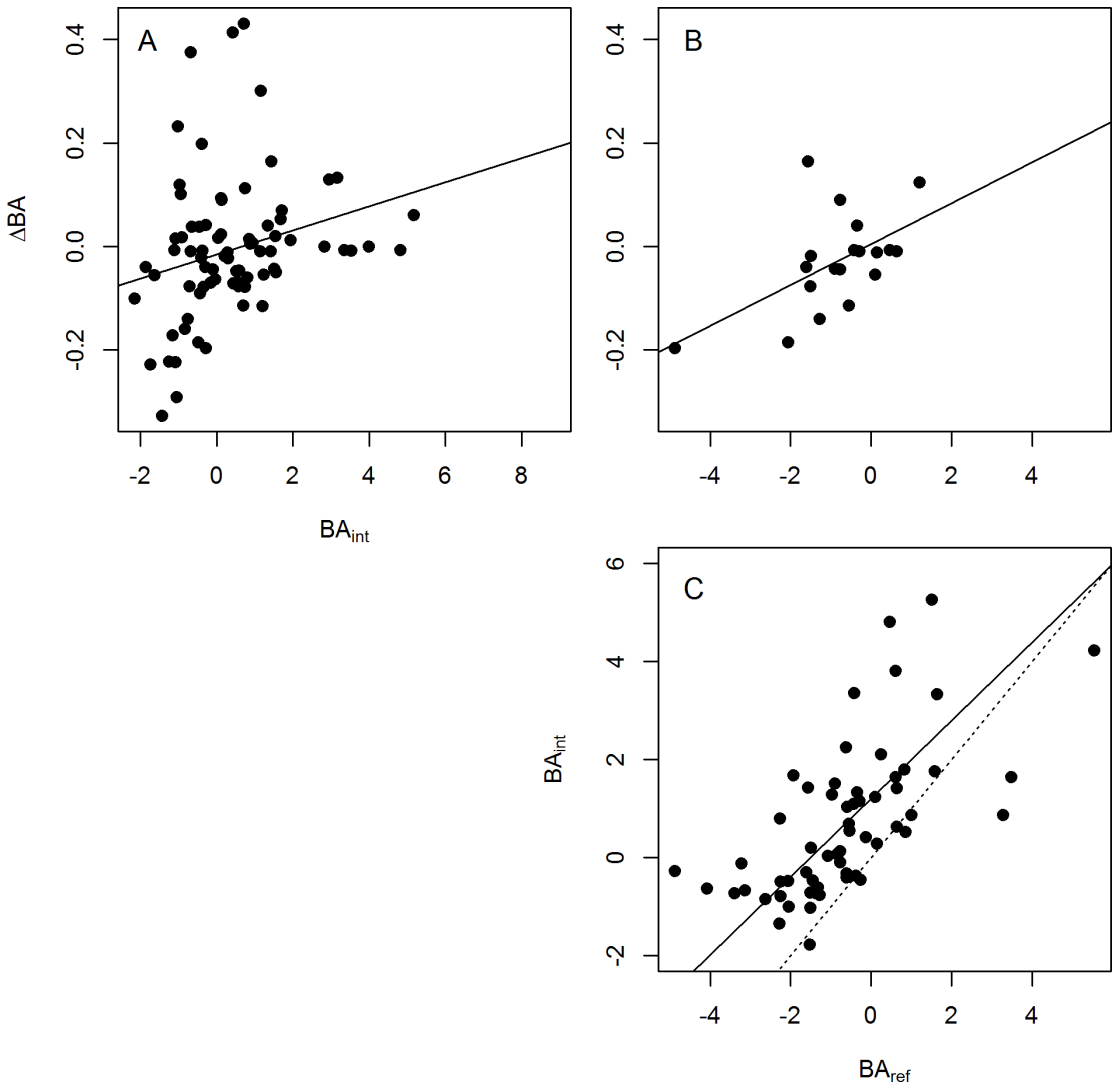
Relationships between basal area variables used in this study. Each point represents a forest-visit. The number of points in each panel is different because not all variables were available for all sites. Solid trend lines indicate a significant (at *p*≤.05) major axis regression. (Note that this fit is different from the more commonly used ordinary least squares regression.) The dotted line in C is a 1∶1 line. A) Annualized basal area change for revisited sites as a function of basal area by regional intercomparison to nearby sites in the database. B) Annualized basal area change for revisited sites as a function of basal area normalized to nearby mature reference forests. C) Basal area regionally intercompared to nearby sites in the database as a function of basal area normalized to nearby mature reference forests.

Basal area (BA) of study forests ranged over several orders of magnitude, from .008 to 97.3 m^2^/ha, (Figures 2A, 3A). Using our new method of regional intercomparisons, most IFRI forests' BA (*BA_int_*) fell within two standard deviations of other nearby IFRI forests, but this variable is a bit over-dispersed (Figure 3B). Comparisons with reference forests had a similar distribution, but with a slightly lower mean, indicating that IFRI forests indeed have less standing stock than nearby old-growth forests. Of the 72 IFRI forests with nearby reference forests, 47 had a BA smaller than or equal to the reference forest mean (Figure 3C). For forests with revisits annualized change in basal area (*ΔBA*) was positively correlated with forest condition measured relative to regional intercomparisons (Figure 4A; *p* = .015, R^2^ = .06) and to reference forests (Figure 4B; *p* = .012, R^2^ = .30).

59 forests had values for BA normalized both to regional intercomparison and to reference forests. With very few exceptions the regional intercomparison z-scores were higher than those relative to reference forests (Figure 4C; note that nearly all points fall above the dotted 1∶1 line). This is to be expected as the reference forests were generally older than the IFRI forests. The relationship between these two variables is clear and positive (*p*<.001, R^2^ = .40).

**Figure 5:**
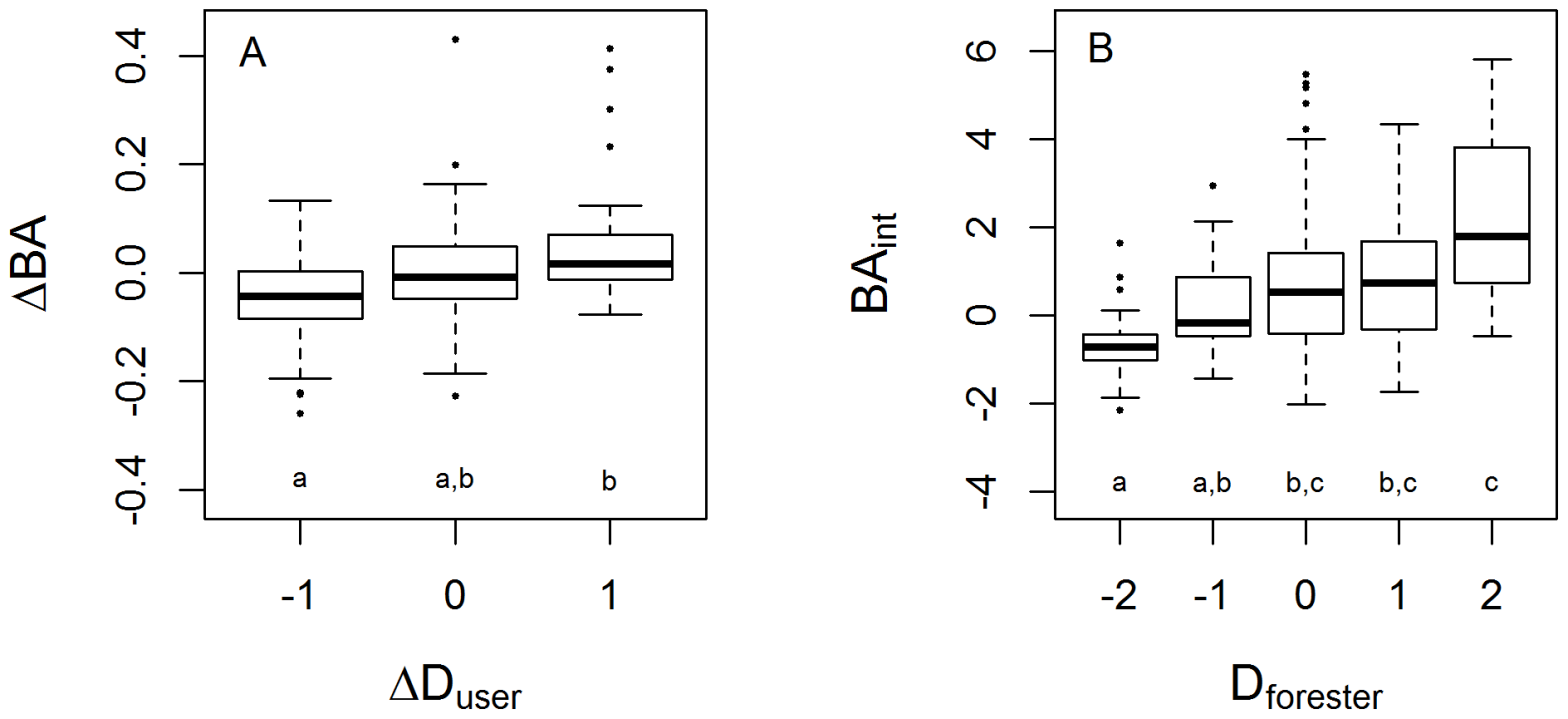
Boxplots of basal area-related variables as estimated ordinally by observers (*ΔD_user_* and *D_forester_*) and as a continuous variable derived from plot-based measurements (*ΔBA* and *BA_int_*). Each point represents a site-visit. Small letters indicate ANOVA-determined significant differences at the *p*≤.05 level using Tukey's honestly significant difference test. A) Annualized change in basal area between site re-visits as determined from plot-based forest surveys (*ΔBA*) vs. forest users' estimates of change in forest density (*ΔD_user_*). “−1” means forest density has decreased, “0” means no change and “1” means forest density has increased. B) Plot-derived forest basal area normalized using regional intercomparison to forests from the same database (*BA_int_*) vs. foresters' estimates of forest density relative to nearby forests (*D_forester_*). Key to category codes: −2: very sparse. −1: somewhat sparse. 0: about normal. 1: somewhat abundant. 2: very abundant.

Users' and experts' qualitative assessments of stand structure were positively correlated with regional intercomparison and forest change over time metrics. For forests thought by users to have an increasing density, *ΔBA* was positive and forests rated as becoming less dense had a negative *ΔBA* on average. These groups were significantly different from one another, although neither could be statistically distinguished from forests estimated by users not to have changed in density (Figure 5A). Similarly, regional intercomparison of plot-measured basal area (*BA_int_*) showed a general correspondence with foresters' ratings of vegetation density compared to regional forests (Figure 5B). In spite of the statistical significance of these results, it is important to note that the variance of plot-based results within each user- or forester-assessed category is large. For user-evaluated forest change more than a quarter of forests rated by users as decreasing in density showed an increase in basal area between site visits and 40% of forests seen by users as increasing in density showed a decrease in basal area. Similarly, nearly one-third of forests rated by foresters as being less dense than average had an above average basal area compared to regional IFRI forests. The converse was also true, with 29% of forests rated by foresters as less dense than average being denser than average in the regional intercomparison (Figure 5).

### Biodiversity

**Figure 6:**
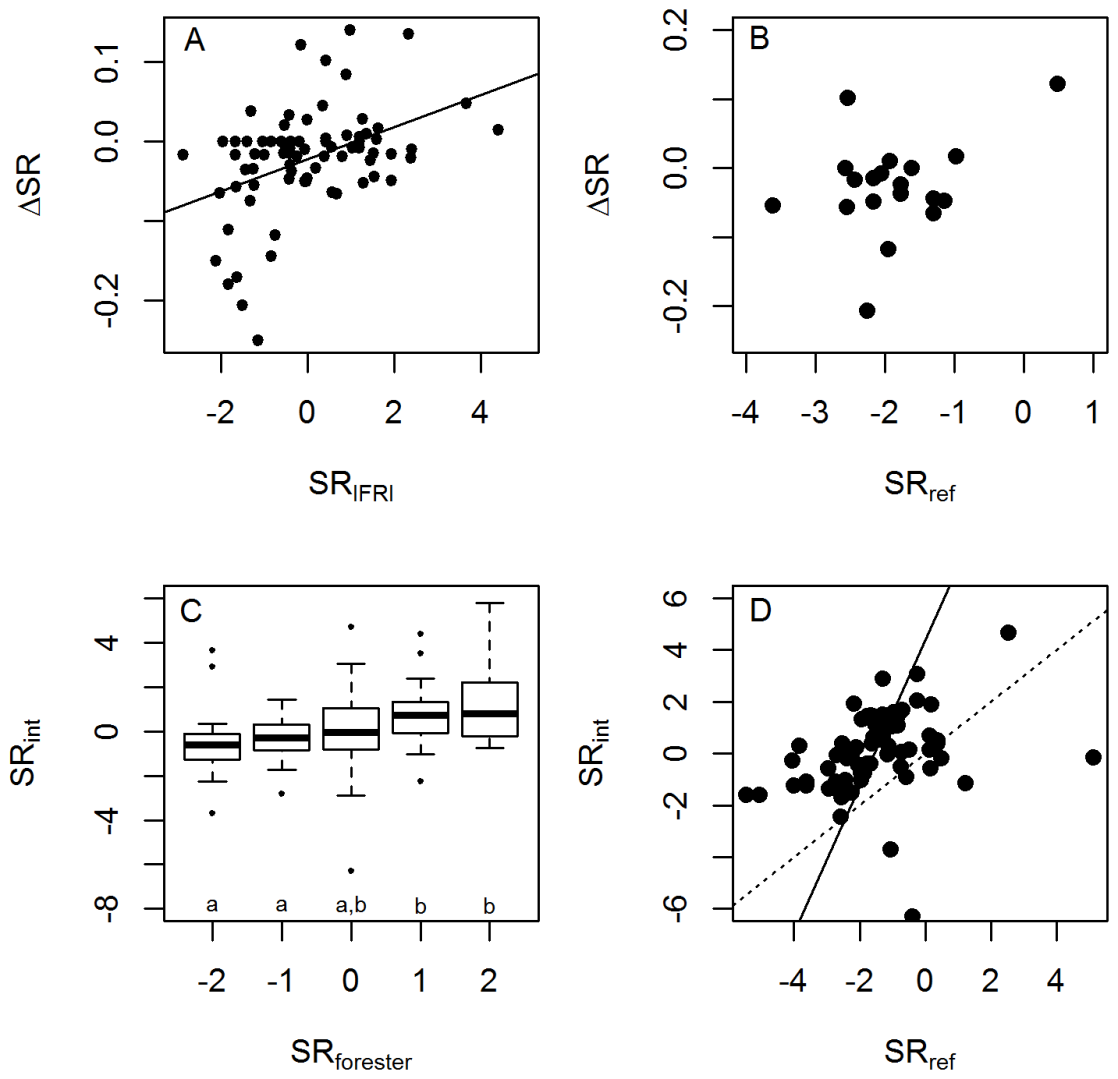
Relationships between biodiversity variables used in this study. Each point represents a forest-visit. The number of points in each panel is different because not all variables were available for all sites. Solid trend lines indicate a significant (*p*<.05) major axis regression (note that this fit is different from the more commonly used ordinary least squares regression.) In part C points are jittered in the x-direction for clarity. The dotted line in D is a 1∶1 line. A) Annualized change in rarefied species richness as determined from plot-based forest samples (*ΔSR*) vs. rarefied species richness normalized by regional intercomparison to forests (*SR_int_*) from the same database. B) Annualized change in rarefied species richness as determined from plot-based forest samples (*ΔSR*) vs. rarefied species richness normalized to nearby mature “reference forests” (*SR_ref_*). C) Rarefied species richness regionally intercompared to forests from the same database (*SR_int_*) vs. foresters' ordinal evaluation of species diversity relative to similar regional forests (*SR_forester_*). Code to categories: −2: very sparse. −1: somewhat sparse. 0: about normal. 1: somewhat abundant. 2: very abundant. D) Species richness regionally intercompared to forests from the same database (*SR_int_*) vs. species richness normalized to nearby reference forests (*SR_ref_*).

Total species richness (SR) of woody stems measured in IFRI plots ranged from 2 to 336 among forests, reflecting the diversity of forest types and sampling intensities among IFRI sites. The median number of species in a sample of 100 stems ranged from 2 to 64 (Figure 1B). Intercomparisons of biodiversity-related variables showed similar patterns to the stand structure variables discussed above. Species richness regionally intercompared with IFRI forests (*SR_int_*), species richness compared to reference forests (*SR_ref_*) and change in rarefied observed species richness (*ΔSR*) were all positively correlated with one another, although the significance of the *ΔSR*-*SR_ref_* relationship (*p* = .068) was slightly above the traditional significance cutoff (Figure 6). Foresters' evaluations of species richness relative to regional forests showed a positive, marginally significant, correspondence with observed species richness in regional intercomparisons (Figure 6C).

### Independence of stand structure and biodiversity measurements

**Figure 7:**
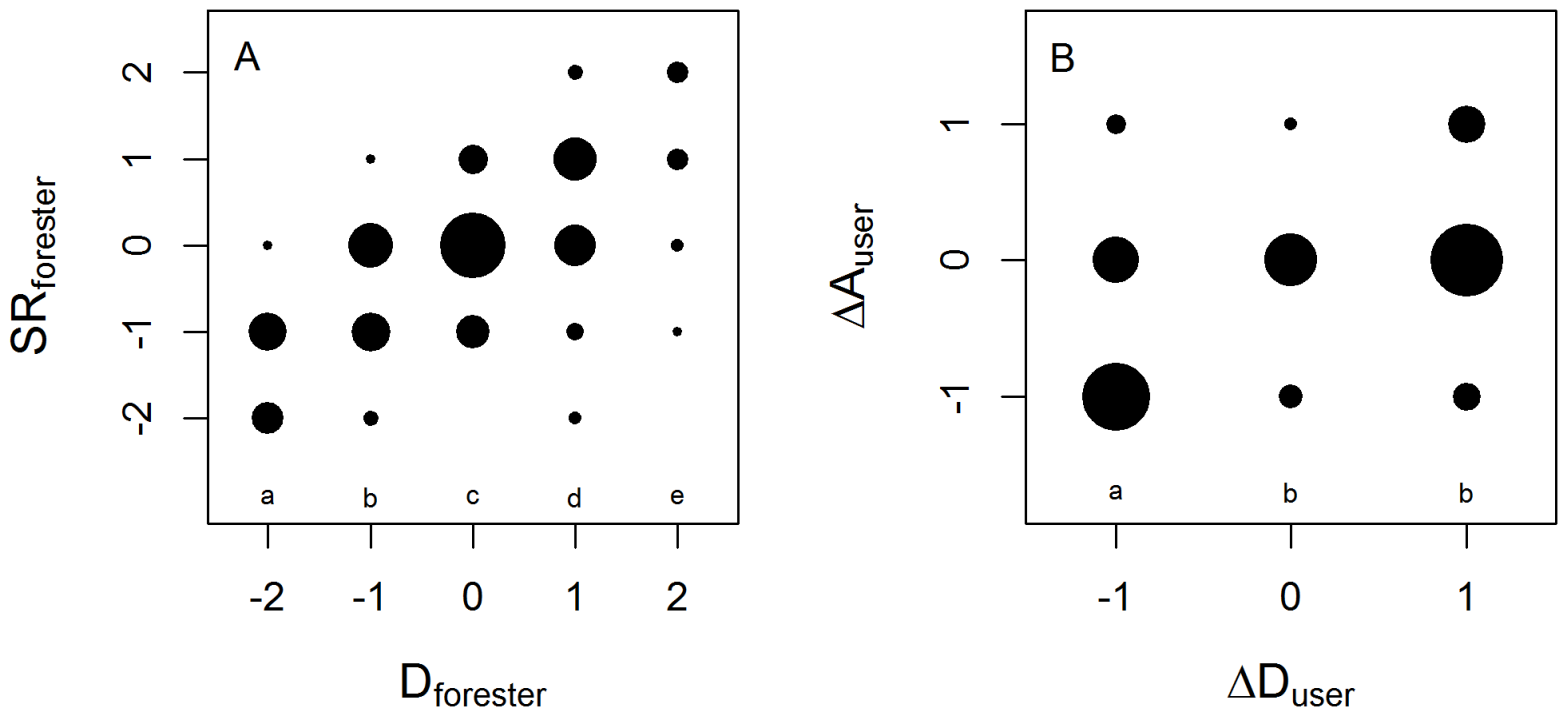
Relationships between different attributes of forests as assessed by the same individuals or groups. For definitions of categories see previous figures. Point area is proportional to the number of sites in the given combination of categories. Small letters indicate ANOVA-determined significant differences at the *p*≤.05 level using Tukey's honestly significant difference test. Because the dependent variable must be treated as continuous for an ANOVA, these analyses were repeated with independent and dependent variables switched, giving virtually identical results. A) Forester-assessed species richness relative to nearby forests (*SR_forester_*) vs. forester-assessed density relative to nearby forests (*D_forester_*). B) User-assessed change in forest area in the last five years (*ΔA_user_*) vs. user-assessed change in forest density in the last five years (*ΔD_user_*).

No correlation was found between most forest structure metrics (i.e., forest density and basal area) and measures of forest area or biodiversity (full results not shown). The only exception to this pattern was forest condition estimates made by the same individual or group. Foresters' estimates of stand density (*D_forester_*) were significantly positively correlated with the same foresters' estimates of species richness (*SR_forester_*), two variables that are usually treated as independent measures of forest condition (Figure 7A). Similarly, forest users' assessments of changes in forest density (*ΔD_user_*) and forest area (*ΔA_user_*) were positively, but less strongly, correlated (Figure 7B).

## Discussion

By comparing forest conditions assessed using a broad suite of methods implemented at nearly 300 community-managed forests in 16 countries, this research has demonstrated that both direct forest measurements and estimates from users and experts have a useful niche in comparative studies of forest management. Comparisons of ground-based measurements of forest change over time and forest condition relative to other reference forests or intercomparison with similarly-managed regional forests can produce broadly congruent results, so long as certain precautions are taken. This conclusion applies to both assessment of stand structure and forest biodiversity. In particular, we have shown that it is not necessary to have “reference forests” or historical baseline data to evaluate the state of forests. Rather, intercomparison with regional forests is shown to deliver comparable results to analysis against reference forests or change over time of individual forests. We have also shown that local users' estimates of forest changes and foresters' comparisons with nearby forests are borne out by direct measurements of forests, although individual estimates can show large deviations from measurements. In addition, qualitative evaluations of different forest attributes such as density and diversity are strongly correlated with one another and may not represent independent aspects of forest condition.

Taken together, these results suggest that researchers have many options to correct for environmental differences in comparative studies of forest outcome. Reference forests and changes between forest revisits remain useful tools when such data is available [21], however in some cases, no such data, or even no such forest, exists. Our results show that when studied forests are geographically clustered, the method of “regional intercomparison” is just as effective as comparison with reference forests. To our knowledge this method has not previously been applied to analysis of forest conditions. Further, regional intercomparisons may lead to a more appropriate measure of forest management success than comparison with “pristine” forests with minimal human impacts. Regional intercomparisons also have an advantage compared to change-over-time methods in that a finding of no change in basal area or species richness could indicate two contrasting scenarios: either a healthy forest that is not being degraded or a young forest under intense harvesting pressure, preventing its growth. Comparison with a regional sample of forests avoids this problem.

While it is encouraging that different methods can provide useful measurements of forests, using caution in their implementation can make them more effective. Here, we highlight several potential pitfalls we have encountered and how they can be avoided. First, while no individual or group can reasonably be expected to have first-hand knowledge of hundreds of sites around the world, data should be reviewed with a skeptical eye and common sense. It was only our initial analysis and questioning of apparent outlying results that led us to uncover the different plot sizes used in some of the sites in India. Second, for biodiversity variables, it is necessary to correct for sampling effort before comparisons are made with reference or other forests. Nagendra [22] assessed a subset of the same data used in this study and found a positive relationship between stem density and per-plot biodiversity. This is to be expected as more stems mean more possible species. Our study has introduced methods of correcting species richness for stem density, increasingly common in the ecological literature [29], but to our knowledge never used in studies of managed forests, further advancing the complex goal of comparing forest conditions in disparate regions. Third, the correlations between qualitative estimates of unrelated variables show that while foresters or forest users may provide useful estimates of forest attributes, it is important to keep in mind that these may actually represent more general, and non-independent, estimates of forest condition.

Related forest metrics (for instance basal area change over time vs. basal area relative to nearby forests) will likely give similar results in broad analyses of forest governance. While some of the measurements analyzed here purport to measure the same phenomenon (user assessments vs. plot assessments of forest density change), others measure different aspects of a forest attribute (basal area change over time within a forest vs. basal area of a forest relative to other forests). In nearly all cases, variables from the same category (i.e. forest structure or species richness) were positively correlated with one another and with users' and foresters' estimates of these variables. This suggests that researchers may have a great deal of latitude in choosing methods for forest analyses and in combining data from different sources that is collected in different ways. However, it is important to bear in mind that the variance within categories of assessment by both foresters and forest users is big. We do not take the view that any particular type of data analyzed here is inherently more reliable than others. Plot-based measurements of necessity do not sample an entire forest and users' or foresters' assessments may be biased in various ways. All of these methods are likely to be incomplete views, particularly for larger forests. This means such assessments are of limited use in case studies or small sample comparisons. If only a few sites are to be compared it is not likely that any of these methods will be definitive, but with sufficient data, a clear and meaningful signal can be extracted from the noise.

Ultimately, the choice among reference forests, regional intercomparisons, over-time forest changes, or users' or experts' evaluations may be driven by resource availability. As noted above, reference forests or baseline data for forest change assessment may not exist. Regional intercomparisons can always be implemented, although obtaining data from enough sites to achieve a useful sample size requires substantial funding and human resources. When research budgets are more limited, experts' and users' evaluations are a viable option, although our results demonstrate that they come at a tradeoff of reduced precision.

While related metrics are basically interchangeable, measurements of different aspects of forests (e.g., biodiversity vs. forest structure variables) are largely independent. Variables from different categories rarely showed any correlation with one another. A prominent exception to this pattern is that individuals' estimates of seemingly unrelated attributes are closely correlated. This is particularly true for foresters' estimates of forest density and species richness relative to nearby forests although less strongly so for local users' estimates of recent changes in forest density and forested area. While there may be an expectation of some relation among these types of variables (to take an extreme example, a recently clear-cut forest would have both a low basal area and low species richness), such strong relationships are not borne out by direct measurements of the forests. It is likely that correlations among individuals' assessments are simply a generalization of an underlying positive or negative view of a particular forest held by the informant, akin to a psychological “halo effect” [32].

“Big data”, such as information repurposed from its original use [33] or crowdsourced from contributors too widely dispersed to efficiently validate [34], imposes new analytical challenges. Direct validation of data quality may be impossible and baselines against which to evaluate outcomes under study can be difficult to define. Although this study has focused on user-managed forests from the IFRI database, the regional intercomparison methods developed here are more broadly applicable. Any multi-locality forest data, such as the expanding network of Center for Tropical Forest Science (CTFS) research forests or national forest inventory programs, lends itself to these methods. Further, the range of questions that could be addressed goes beyond forest management to phenomena like climate change or invasive insect impacts. This paper has shown a way forward in the face of this type of problem.
